# Newborn Screening: Review of its Impact for Cystinosis

**DOI:** 10.3390/cells11071109

**Published:** 2022-03-25

**Authors:** Katharina Hohenfellner, Ewa Elenberg, Gema Ariceta, Galina Nesterova, Neveen A. Soliman, Rezan Topaloglu

**Affiliations:** 1 Department of Pediatric Nephrology, RoMed Clinis, Pettenkoferstr. 10, 83022 Rosenheim, Germany; 2 Department of Pediatrics, Texas Children’s Hospital, Baylor College of Medicine, 6701 Fannin Street 11th Floor, Houston, TX 77030, USA; exelenbe@texaschildrens.org; 3 Department of Pediatric Nephrology, University Hospital Vall d’ Hebron, University Autonomous Barcelona, Passeig de la Vall d’Hebron, 119-129, 08035 Barcelona, Spain; gariceta@vhebron.net; 4 Cystinosis Research Network, Medical Advisory Committee, Chicago, IL 60045, USA; gnesterova@hotmail.com; 5 Department of Pediatrics, Center of Pediatric Nephrology and Transplantation (CPNT), Kasr Al Ainy Faculty of Medicine, Cairo University, 99 El-Manial Street, Cairo 11451, Egypt; neveenase@yahoo.com; 6 Department of Pediatric Nephrology, Hacettepe University School of Medicine, Ankara 06610, Turkey; rezantopaloglu@hacettepe.edu.tr

**Keywords:** newborn screening, infantile nephropathic cystinosis, clinical course, *CTNS*-pathogenic variants, newborn screening for cystinosis

## Abstract

Newborn screening (NBS) programmes are considered to be one of the most successful secondary prevention measures in childhood to prevent or reduce morbidity and/or mortality via early disease identification and subsequent initiation of therapy. However, while many rare diseases can now be detected at an early stage using appropriate diagnostics, the introduction of a new target disease requires a detailed analysis of the entire screening process, including a robust scientific background, analytics, information technology, and logistics. In addition, ethics, financing, and the required medical measures need to be considered to allow the benefits of screening to be evaluated at a higher level than its potential harm. Infantile nephropathic cystinosis (INC) is a very rare lysosomal metabolic disorder. With the introduction of cysteamine therapy in the early 1980s and the possibility of renal replacement therapy in infancy, patients with cystinosis can now reach adulthood. Early diagnosis of cystinosis remains important as this enables initiation of cysteamine at the earliest opportunity to support renal and patient survival. Using molecular technologies, the feasibility of screening for cystinosis has been demonstrated in a pilot project. This review aims to provide insight into NBS and discuss its importance for nephropathic cystinosis using molecular technologies.

## 1. Introduction

Newborn screening programmes (NBS) aim to identify presymptomatic newborns with rare serious or fatal disorders that can be successfully treated, thereby achieving a significant reduction in morbidity and mortality [1]. NBS programmes are now implemented in more than 50 countries worldwide [1].

NBS represents a mostly underestimated, complex process involving different representatives of the public healthcare system to enable sample collection, analysis, communication of the result and, if necessary, coordination of treatment initiation, within a narrow time window [2,3]. Accordingly, there are high demands on the process quality of an NBS programme in terms of communication, logistics, analysis, and evaluation.

NBS programmes are country-specific and dependent on their respective healthcare systems, with their associated socio-economic, cultural, ethical, and legal requirements [1,4,5,6,7]. Before introducing screening or adding another target disease, the benefits (reduction in morbidity and mortality), harms (overdiagnosis, false positives, false negatives), and costs incurred during the screening process (implementation of screening and subsequent treatment costs) have to be assessed, taking into account available resources (human, analytics, information systems) and ethical aspects [2,8,9,10,11].

The history of NBS is closely linked to the fate of Dr Robert Guthrie. In the early 1950s, Horst Bickel demonstrated that the implementation of a low-phenylalanine diet from an early age could prevent the mental retardation associated with phenylketonuria (PKU) [12]. While the diagnosis of PKU in the early 1960s utilized the presence of phenylpyruvic acid in the urine, the sensitivity of this method was not sufficient enough to identify patients prior to the development of irreversible brain damage [13]. Following bacteriological tests used in cancer research, Dr Guthrie developed a test that could be used as a screening method for PKU [14]. In 1961, the first screening of newborns began, and by 1965 all newborns had been screened for PKU in the USA [15]. In the following years, a programme of NBS concerning PKU was established in other countries and was successively extended to include other diseases, due to the availability of newer technologies (radioimmunoassay, colorimetric and fluorometric assays, and enzymatic, isoelectric focusing, high-performance liquid-chromatography) [16]. The methodological limitation of NBS in the mid-1990s was the number of individual tests required for the various diseases. With the introduction of tandem mass spectrometry (MS/MS), which enables the cost-effective simultaneous identification of up to 50 metabolic conditions from amino acid metabolism, organic acid metabolism, and fatty acid degradation from a single blood spot, this limitation could be overcome [17]. Several new additions to NBS programmes, such as those for cystic fibrosis [18], severe combined immunodeficiency (SCID) [19], and spinal muscular atrophy (SMA) [20], have been possible following the introduction of molecular technologies.

The screening framework was formulated by Wilson and Jungner in 1968 under the auspices of the World Health Organization (WHO) and is still valid today in an adapted form [21,22]. According to these principles, each target disease requires clarification of sufficient available knowledge, and it needs to represent a relevant health problem for the individual/population group which cannot be diagnosed clinically with certainty in the neonatal period. In addition, appropriate therapy must be available, with early initiation of treatment resulting in reduced morbidity and mortality. The screening test procedure should display high sensitivity and specificity, be cost-effective, and any benefit/harm assessment of test must be on the side of ‘benefit’ [21] (Table 1).

**Figure 1:**
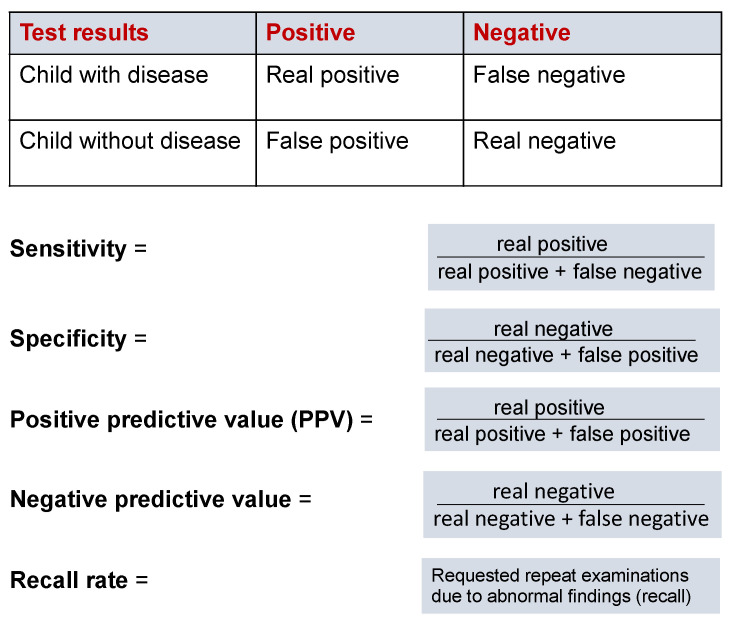
Test results and quality parameters in neonatal screening. Sensitivity: Ability of the test to accurately identify those individuals with a specific condition/disease. Specificity: Ability of the test to accurately identify those individuals without the condition/disease. Positive predictive value (PPV): Probability that the person tested has the disease when the test is positive. Negative predictive value: Probability that the person does not have the disease, when the test is negative.

NBS involves examining a large population of clinically inconspicuous newborns in order to identify very few affected children [3,23,24]. Of note, 99.9% of all children examined in NBS programmes are found to be without any noticeable finding [3,23,24]. As such, this necessitates the use of a subsequent stepwise analytical approach. The initial analytical test method must have a high sensitivity and specificity and should be able to identify or exclude the disease with the highest possible certainty (Figure 1) [23]. If the screening result is positive, the individual is not necessarily ill, but does require a more disease-specific workup. The recall rate describes the percentage of control examinations due to a conspicuous finding in the initial screening. The positive predictive value indicates the probability of actually having the disease in the case of a positive test result, depending on the prevalence of the target disease and the specificity of the chosen method.

From a diagnostic perspective, the aim of NBS is to increase the specificity of the analysis and to identify individuals with mild phenotypes, i.e., carriers not requiring treatment, thus reducing costs and the psychological burden on the parents caused by unnecessary further examinations. The notification of a positive finding always causes a high burden for the family [25,26]. Parents of children with a false-positive screening result for a metabolic genetic disease have a 23% higher stress level [25], while a 143% increase in depressive mood has been reported in parents with false-positive results for cystic fibrosis [26].

So far, NBS programmes have demonstrated both substantial benefits and deficits, preventing the full benefit of NBS from being achieved [27,28]. Only a few screening programmes, such as those in Sweden or the German state of Bavaria, include matching with a birth registry and allow completeness of coverage to be verified [29]. In addition, despite considerable effort and expense at all levels, tracking systems that follow up positive screenings to verify that controls and/or confirmation diagnostics have been performed and therapy has been initiated are not available in most countries. Long-term systematic monitoring (clinical and laboratory parameters) of diagnosed patients identified by screening is also not ensured [30]. The lack of feedback from or recording of those patients not identified during the screening process remains largely unregulated, preventing any valid and robust statement being made on the sensitivity of NBS. Considering the rarity of most diseases in any newborn panel, there is an ongoing need to combine all available data to provide a clearer clinical picture on confirmed genotype/phenotype correlation and the different expression of the diseases, age of disease onset, and treatment response.

Regarding the target diseases, there are significant differences even within Western countries [5,31]. NBS programmes in European Union (EU) member states differ significantly in terms of target diseases, among other factors [5]. NBS in France, Germany, and Austria includes 9, 17, and 26 target diseases, respectively [5]. There are no current policy recommendations or direct NBS overview at a European level as the sole responsibility for setting health policy and managing health/medical care, including financing of services and overall scope, lies with the respective member states. Harmonization of NBS programmes for target diseases at a European level is a current request of the European patient organization EURORDIS [32,33]. Central efforts to standardize NBS have been underway in the USA since 2000 [34,35,36]. With the help of the American Academy of Pediatrics Task Force, the Advisory Committee on Heritable Disorders in Newborn and Children (ACHDNC) was established. The American College of Medical Genetics (ACMG) created an evidence-based core screening panel in 2005, which was subsequently adopted as the recommended uniform screening panel by the ACHDNC and recommended by the Secretary of Human Services [31,37]. This screening panel contains 35 core conditions and 29 secondary conditions (differential diagnostic features) which have been adopted and implemented by almost all US states.

## 2. Cystinosis

Cystinosis is a rare autosomal recessive systemic disease with high morbidity and mortality caused by pathogenic variants in the *CTNS* gene that encodes the lysosomal cystine transporter cystinosin, leading to accumulation of cystine within the lysosome [38,39]. Life-long cystine-depleting therapy with oral cysteamine, the only specific therapy for cystinosis, along with the availability of renal replacement therapy in childhood, has dramatically improved patient outcomes [40]. There is robust evidence that early initiation and sustained therapy with cysteamine are both essential for delaying progression to chronic kidney disease (CKD) and end-organ damage [41].

Infantile nephropathic cystinosis is the most common cause of renal Fanconi syndrome in children and is a hallmark of the disease [42]. Renal Fanconi syndrome presents with proximal renal tubular acidosis along with a generalized dysfunction of the proximal tubule, characterized by the presence of polyuria, glycosuria, phosphaturia, tubular proteinuria, growth retardation, and rickets; later glomerular involvement leads to progressive kidney failure. The proximal tubular cells (PTCs) are first to be affected [43]. However, evidence from mouse models suggests that differentiation (structural changes) of PTCs starts prior to the accumulation of cystine crystals in both PTCs and the interstitium, leading to a loss of their brush border, flattening and thickening of tubular basement membrane, and the eventual development of the characteristic swan-neck deformity [44]. These changes progress to tubular atrophy and, in addition, heavy inflammatory cell infiltrates can be observed in the renal interstitium. Glomerular involvement with multinucleated podocytes and focal segmental glomerulosclerosis lesions can be seen in renal biopsies [42]. While the defect in cystine transport by cystinosin is the hallmark of cystinosis, it is not the only key player in the pathogenesis of renal Fanconi syndrome; cystinosin has additional roles, including regulation of autophagy, mTOR signalling, lysosomal biogenesis, and vesicle trafficking in proximal tubular epithelial cells.

Early diagnosis of cystinosis enables treatment with cysteamine, the only specific therapy for the disease, which should be administered as early as possible and continued throughout the life of the patient [38]. It is well accepted that early treatment with cysteamine improves patient outcome, delays progression to renal failure with a mean age of 9 years when starting dialysis, and prevents or attenuates end-organ damage [41,45,46,47,48,49]. Initiation of cysteamine before 3, 2.5, or even <2 years of age has been associated with preservation of renal function in patients with cystinosis [40,45,46,47,50,51,52]; thus, patient age at initiation of cysteamine therapy appears to be a major predictive factor of renal survival. In a large international contemporary cohort of 453 patients with cystinosis, cysteamine was initiated in 89% of patients at a median age of 1.6 years, and a near linear relationship between the age of cysteamine initiation and renal function was observed [47]; patients treated before the age of 1 year exhibited the best renal outcome. All these findings provide the rationale to develop NBS to diagnose cystinosis as early as possible, ideally before the development of clinical manifestations and irreversible PTC damage prior to cystine crystal accumulation. Table 2 summarizes published evidence (in chronological order) of the impact of early treatment with cysteamine on renal outcome in patients with cystinosis [53].

Progressive renal failure develops in most patients with nephropathic cystinosis [41,55]. Renal transplantation is the best therapeutic option for end-stage renal disease and improves both survival rate and quality of life [56]. More pre-emptive transplants in patients with cystinosis reflect temporal changes in paediatric transplantation practice [57]. Mortality in patients with cystinosis who undergo transplantation occurs at a late stage (10 years post-transplantation), frequently from extrarenal manifestations of cystinosis, and contrasts with a relatively good 5-year post-transplantation survival. Renal transplantation markedly improves the lifespan of patients with cystinosis, although cystine accumulation continues in non-renal organs [46]. While Fanconi syndrome does not recur in the transplanted organ, cysteamine therapy needs be continued for the lifetime of the patient to prevent extrarenal complications.

Beside time of diagnosis, adherence to cysteamine therapy is another critical factor for preservation of renal function [58]. The benefits of long-term cysteamine therapy are most evident in patients with good adherence to treatment.

## 3. Prenatal Testing and Preimplantation Genetic Diagnosis

Families known to be at risk of INC can consider options such as preimplantation genetic diagnosis or prenatal diagnosis [59]. There are two options for prenatal testing: biochemical testing and molecular genetic testing [59]. For pregnancies at risk of INC, prenatal diagnosis is possible biochemically, by measuring ^35^S-labeled cystine accumulation in cultured amniocytes (14–16 weeks of gestation) or chorionic villi samples (CVS) (8–9 weeks of gestation) and by a direct measurement of cystine in uncultured CVS [60]. Once the *CTNS* pathogenic variants have been identified in an affected family member, for a pregnancy at increased risk and preimplantation genetic diagnosis for cystinosis are possible. Differences in perspective may exist among medical professionals and within families regarding the use of prenatal testing, particularly where this is being considered for the purpose of pregnancy termination rather than early diagnosis of a target disease. DNA analysis for detecting mutant alleles is currently the most frequently used antenatal screening method. While most clinical centres typically consider decisions regarding prenatal testing to be the parents’ choice, discussion of any potential issues with the medical team remains important, together with the possible involvement of a genetic counsellor.

## 4. CTNS Pathogenic Variants in Different Countries

The *CTNS* gene for cystinosis, which encodes cystinosin, was identified by Town et al. in 1998 [39]. Since then, approximately 150 *CTNS* pathogenic variants have been described in patients with cystinosis worldwide [61]. The first pathogenic variant of the *CTNS* gene was reported as a large 57-kb deletion involving the first 9 exons and part of exon 10 of the *CTNS* gene, along with two upstream genes (*CARKL* and *TRPV1*). The 57-kb deletion is the most common pathogenic variant that causes cystinosis in Northern Europe and North America [62,63]. Germany is considered to be the country of origin for the 57-kb deletion which now accounts for 50–70% of the alleles in the USA and Northern Europe [63,64]. The frequency of the 57-kb deletion in patients with cystinosis is 22% in Mexico, 17% in Italy, and 0% in Turkey and Egypt [49,65,66,67].

A single-centre Turkish study and a Turkish national study based on the national registry of cystinosis confirmed that the most common allele (31%) in Turkey is c.681G>A (p.E227E), a missense pathogenic variant that causes a frameshift [49,68]. In Iran, the most common pathogenic variant is c.681G>A (p.E227E) [69]. Studies from Egypt show no patients with cystinosis having the 57-kb deletion, but they have confirmed that the c.829dup (p.T277NfsX19) is the most common *CTNS* pathogenic variant identified in a homozygous state among Egyptian patients with cystinosis [67]; this pathogenic variant has been reported only once in a heterozygous state in a European patient. The exclusive identification of c.681G>A (p.E227E) pathogenic variant, with variable frequencies, in the Middle Eastern population, along with its absence in European and American populations, suggests the existence of a possible founder pathogenic variant in this area.

c.1015G>A (G339R) has been identified as a founder pathogenic variant for cystinosis in the Ontario Amish Mennonite population in Canada [63]. This missense pathogenic variant has also been frequently observed in patients with cystinosis in Northern and Southern Italy, Spain, Turkey, and the Middle East [61,68]. Another possible founder pathogenic variant is c.971-12G>A which has been identified in the black population of South Africa [70]. A further pathogenic variant of interest is the nonsense stop codon pathogenic variant p.W138X, which accounts for 50% of cystinotic alleles in the French-Canadian population [71]. This novel stop codon pathogenic variant shows the possibility for read through in the presence of an aminoglycoside, which may enable a potential treatment.

The impact of the various types of *CTNS* pathogenic variants has been investigated and it is thought that individuals who harbour severe pathogenic variants, such as loss of function pathogenic variants on both alleles, have severe infantile cystinosis, while individuals homozygous or compound heterozygous for milder pathogenic variants have milder forms of the disease [41,63,72]. Homozygosity for the 57-kb deletion is associated with an increased risk of morbidity and mortality [41].

Of note, recent studies have shown that pathogenic variants appear to have no significant impact on either kidney function or progression to kidney failure in infantile cystinosis, whereas early initiation of cysteamine treatment has a significant impact on the preservation of renal function [47,49].

## 5. Newborn Screening for Cystinosis

The implementation of genetic testing in NBS was undertaken in Utah in 1998 for the detection of sickle cell disease [73]. Molecular biological methods have since been introduced as first tier, second tier, or third tier methods in NBS [74].

Currently, the diagnosis of cystinosis is based on the presence of elevated cystine levels in white blood cells [39]; this method is unsuitable for NBS. In 2018, as part of a pilot study, the existing German NBS programme was expanded to incorporate high-throughput first-tier molecular genetic screening for cystinosis and spinal muscular atrophy (SMA) [75,76]. Both congenital disorders are suitable for molecular-based NBS because they have known genetic causes and effective therapies are available. Based on the results of the pilot project, SMA screening has been successfully included as a regular part of NBS in Germany [77].

For cystinosis screening in Germany, the first tier involved multiplex PCR to detect the three most common *CTNS* pathogenic variants, i.e., a 57 kb deletion, c.18_21delGACT, p.T7Ffs*7, and c.926dupG, p.S310Qfs*55 [64]. Heterozygous samples were submitted to amplicon-based next-generation sequencing for 101 pathogenic *CTNS* pathogenic variants published at the time. A detection rate of 98.5% was subsequently predicted using this approach [75]. In 299,631 newborns, two patients with a homozygous 57-kb pathogenic variant and one patient with a 57-kb compound homozygous pathogenic variant were identified. A total of 805 patients with heterozygous pathogenic variants were identified, 655 with 57-kb pathogenic variant, 85 with c.18_21delGACT, p.T7Ffs*7, and 65 with c.926dupG, p.S310Qfs*55.

In the first patient identified with a homozygous 57-kb pathogenic variant and confirmed diagnosis by determining the leucocyte cystine level (2.82 nmol cystine/mg protein; normal, <0.2), treatment was initiated within the first month of life [78]. Even at the age of 3.5 years, the patient presented with normal physical development (height 20% percentile, weight 21% percentile) and without renal Fanconi syndrome or proteinuria. Apart from cysteamine treatment, the patient did not require any other pharmaceutical therapy. Unfortunately, the mother of the second patient with a homozygous 57-kb deletion initially refused NBS for cystinosis. At 8 months of age, the child was admitted to an intensive care unit with a severe electrolytic disturbance. In the course of the latter, a dried blood (DBS) card was re-sent to the screening laboratory and re-evaluated to include cystinosis, which yielded a positive test result. The patient then presented with the full clinical presentation of Fanconi syndrome and required high electrolyte replacement and growth hormone therapy in addition to cysteamine therapy. A third neonate, screened as heterozygous for the common 57-kb deletion, was found to harbour an additional promotor variant (c.-512G>C) in *CTNS* previously reported as being disease causing. However, according to current ACMG schemes, the respective promotor variant needs to be reclassified as a non-pathogenic change [78]. In fact, this infant showed no biochemical evidence of cystinosis, with a normal leucocyte cystine level, i.e., <0.2 nmol cystine/mg protein).

Detection rates estimate the known incidence of cystinosis at (1:150,000–1:200,000) [79]. False positive and false negative results did not occur until now. One key requirement of all NBS programmes is that they must provide direct clinical benefit [21]. Patients with cystinosis are generally diagnosed at 12–18 months of age, by which time significant renal tubular and glomerular damage has already occurred [58,80]. As previously described, early treatment with oral cysteamine has salutary effects on preservation of renal function, growth, and prevention of late complications of the disease [58]. For those few infants treated shortly after birth due to an older sibling already having cystinosis, even the renal tubular Fanconi syndrome that typically presents in the first months of life was ameliorated in these individuals [40].

Molecular screening for the 57-kb pathogenic variant can be combined with an existing NBS for severe combined immunodeficiency (SCID) and/or SMA. According to the results of the German pilot project, 655 of approximately 300,000 newborns carried a heterozygous 57-kb pathogenic variant, which required further screening with NGS for defined *CTNS*-pathogenic variants [78]. The feasibility of implementing NGS in a regular NBS programme has been demonstrated in Norway, where NBS was increased from 2 diseases to 25 in 2018 [24], using a second-tier strategy utilizing MS/MS methodologies and NGS for certain diseases. Thus, screening in Northern Europe and North America for the 57-kb pathogenic variant homozygous and heterozygous with downstream NGS, where the 57-kb allele accounts for 50–70% of the alleles, appears to be feasible [63,64]; this is further supported by the fact that also in other target diseases not all patients are identified (e.g., late-onset hypothyroidism and atypical adrenal hyperplasia).

Due to the heterogeneity of screening panels in different countries, the often slow and difficult implementation of additional target diseases, and the limited availability of genomic sequencing in public health and clinical settings, commercial laboratories have begun to offer genomic screening panels for newborns. Hopefully, these will not be as successful as the commercial tests already available from Ancestry, 23andMe, or MyHeritage, whose 2019 databases were estimated at 20 million, 12 million, and 2.5 million, respectively [81,82].

## 6. Conclusions

NBS programmes are secondary prevention measures in early childhood to prevent or reduce morbidity and/or mortality via early disease identification and subsequent initiation of therapy. Specific target diseases, and thus screening programs, may vary from country to country.

In cystinosis, both diagnosis and treatment at the earliest possible stage is the clinical goal. There is great hope that the use of neonatal testing to enable this early diagnosis and treatment, as discussed in this article, will provide a turning point in the natural history of cystinosis.

Given that more than 150 *CTNS* pathogenic variants have been described, countries/continents could implement NBS for the most commonly reported ones. For Northern Europe and North America, screening for the 57-kb deletion would prove useful and this could be augmented by other commonly reported pathogenic variants. However, this approach may not be possible in all countries, particularly those with limited resources and no known founder pathogenic variant.

In the future, we will have advanced packages for NBS, as early diagnosis and treatment of diseases will gain even more importance. In addition, other methods for early detection may be possible, such as preimplantation genetics and in vitro fertilization in families with known risks.

## Figures and Tables

**Table 1:** Principles of screening by Wilson and Jungner (1968).

1.	The condition sought should be an important health problem.
2.	There should be an accepted treatment for patients with recognized disease.
3.	Facilities for diagnosis and treatment should be available.
4.	There should be a recognizable latent or early symptomatic stage.
5.	There should be a suitable test or examination.
6.	The test should be acceptable to the population.
7.	The natural history of the condition, including development from latent to declared disease, should be adequately understood.
8.	There should be an agreed policy on whom to treat as patients.
9.	The cost of case finding (including diagnosis and treatment of patients diagnosed) should be economically balanced in relation to possible expenditure on medical care as a whole.
10.	Case finding should be a continuing process and not a “once and for all” project.

**Table 2:** Impact of early initiation of oral cysteamine.

First Author, Year	Study Description	Impact of Early Initiation of Cysteamine on Renal Function	Impact of Early Initiation of Cysteamine on Extra-Renal Manifestations
Kleta, 2004 [40]	Family case report of two siblings (children)	A non-symptomatic child, identified after an affected brother who initiated cysteamine at 2 months of age, achieved and maintained normal renal function at 8 years of age. Patient’s brother had CKD stage II at similar age despite treatment with cysteamine from 2 years of age	NR
Broyer, 2008 [50]	Necker Enfants-Malades Hospital series; patients born before 1988; aged 20–39 years (n = 56)	Initiation < 3 years of age vs. later delayed ESRD onset (mean age at onset 17.4 vs. 9.6 years)	Initiation < 3 years of age vs. later: Improved linear growth and visual acuity Reduced rates of glucose intolerance, thyroxine requirements, myopathy, cerebellar/pyramidal symptoms or mental deterioration, and hepatosplenic disorders
Greco, 2010 [45]	Italian single-centre study; patients diagnosed at 3–60 years of age; median follow-up 17.6 years (n = 23)	Initiation < 2.5 years of age vs. later improved evolution of renal function (*p* = 0.006), and decreased risk of CKD stage III	Patients treated more recently (initiated < 2.5 years of age) had improved linear growth curves vs. older children
Vaisbich, 2010 [51]	Brazilian multicentre nephropathic study; patients aged 1.3–29 years enrolled since 1999 (n = 102)	Initiation < 2 years of age vs. later reduced rate of CKD stage II–V (25% vs. 77.5%)	Initiation < 2 years of age vs. later: Improved growth (weight and height) parameters Reduced rates of hypothyroidism, diabetes, muscular weakness, hepatic dysfunction, CNS disorders, and swallowing dysfunction
Brodin-Sartorius, 2012 [46]	French study; adults (aged ≥15 years) diagnosed between 1961 and 1995 (n = 86)	Initiation < 5 years of age vs. later delayed ESRD onset (mean age at onset 13.4 vs. 9.6 years; *p* < 0.05)	Initiation < 5 years of age (vs. later or no treatment): reduced rates of hypothyroidism, diabetes, neuromuscular disorders, and death
Viltz, 2013 [54]	Children and adolescents (aged 3–18 years) [n = 46]	NR	Initiation < 2 years of age vs. later: improved cognitive function (verbal, performance, and full-scale IQ scores, and spatial-relations test), but not visual-motor performance scores
Bertholet-Thomas, 2017 [52]	Multinational study in children and adolescents from 41 centres and 30 nations (n = 213)	Earlier cysteamine treatment resulted in better renal outcome. Median renal survival increased up to 16.1 (12.5-/) years in patients treated at <2.5 years of age (*p* = 0.0001)	NR
Topaloglu, 2017 [49]	Multicentral study in children and adolescent from 26 centres [n = 136]	Patients in whom cysteamine treatment was initiated at age < 2 years old had delayed progression to renal failure compared to the patients in whom cysteamine treatment was initiated > 2 years (*p* = 0.02)	NR
Emma, 2021 [47]	Large international cohort of patients followed along five decades (n = 453)	A nearly linear relationship between the age at cysteamine initiation and renal survival was observed. Patients who started cysteamine aged < 1 year had delayed progression to renal failure in comparison with those who started aged 1–2 years old, and those who started cysteamine after 2 years of age (HR: 1.24; 95% CI: 1.9, 1.42; *p* < 0.002)	Initiation of cysteamine before the age of 1.5 years had a positive effect on growth with a gain of 0.57 SDSs in comparison with those who started cysteamine later.

CI, confidence interval; CKD, chronic kidney disease; CNS, central nervous system; ESRD, end-stage renal disease; HR, hazard ratio; NR, not referred; SDS, standard deviation score.
